# Only one beer can be mortal: a case report of two sisters with cardiac arrest due to a homozygous mutation in *PPA2* gene

**DOI:** 10.1007/s00431-023-05034-9

**Published:** 2023-06-03

**Authors:** Héctor Hugo Manzanilla-Romero, Elisabeth Schermer, Agnes Mayr, Sabine Rudnik-Schöneborn

**Affiliations:** 1 Institute of Human Genetics, Medical University of Innsbruck, Innsbruck, Austria; 2 Pediatrics III (Cardiopulmonary Unit), Medical University of Innsbruck, Innsbruck, Austria; 3 Clinic of Radiology, Medical University of Innsbruck, Innsbruck, Austria

**Keywords:** Alcohol, Mitochondriopathy, Dilated cardiomyopathy, Sudden cardiac death, Exome sequencing

## Abstract

We report the long way to the correct diagnosis in two teenage sisters who developed a cardiac arrest after consuming minimal amounts of alcohol. The older girl dramatically survived two cardiac arrests at the age of 14 and 15 years. She underwent an extensive examination that revealed isolated cardiac abnormalities including fibrosis, dilated cardiomyopathy and inflammation. The younger girl also had a cardiac arrest at the age of 15 and died suddenly after consuming 1–2 beers, 3 years after her sister´s first incident. Autopsy of the heart revealed acute myocarditis without structural alterations. Multigene panel analysis (not including *PPA2*) showed *SCN5A* and *CACNA1D* variants in both sisters and their healthy mother. Six years later duo exome allowed the diagnosis of an autosomal recessive PPA2-related mitochondriopathy. We discuss the molecular results and clinical picture of our patients compared to other PPA2-related cases. We highlight the diagnostic contribution of multigene panels and exome analysis. The genetic diagnosis is important for medical care and for everyday life, specifically because alcohol intake can result in cardiac arrest and should be strictly avoided.

*Conclusion*: Duo exome sequencing clarified the diagnosis of PPA2-related mitochondriopathy in two sisters with isolated cardiac features and sudden cardiac arrest triggered by minimal amounts of alcohol.

**Table:** 

**What is Known:** *• Multigene-Panel or exome analysis is a valuable tool to identify genetic causes of hereditary cardiac arrhythmias.* *• Variants of unknown significance can lead to misinterpretation. PPA2-related mitochondriopathy is a very rare autosomal recessive condition that is normally fatal in infancy.*	
**What is New:** *• Duo exome analysis in two teeenage sisters with cardiac arrest revealed a homozygous mild PPA2 mutation as the underlying pathology restricted to the heart muscle*

**What is Known:**

*• Multigene-Panel or exome analysis is a valuable tool to identify genetic causes of hereditary cardiac arrhythmias.*

*• Variants of unknown significance can lead to misinterpretation. PPA2-related mitochondriopathy is a very rare autosomal recessive condition that is normally fatal in infancy.*

**What is New:**

*• Duo exome analysis in two teeenage sisters with cardiac arrest revealed a homozygous mild PPA2 mutation as the underlying pathology restricted to the heart muscle*

## Introduction

Sudden unexpected death in the young occurs 1–3 to 100′000 individuals in Europe every year. Thirty to 40% of the autopsies remain inconclusive after conventional procedures including histology, immunochemistry, and toxicology. Thereafter, postmortem genetic testing through next-generation sequencing (NGS) techniques may detect a plausible origin [1].

Mitochondriopathies are a vast group of genetic diseases that manifest at different stages of life with a wide spectrum of clinical expressions. First published in 2016 [2, 3], the deficiency of the mitochondrial enzyme pyrophophastase type 2 (PPA2) is a rare autosomal recessive disease that leads to sudden cardiac death in young patients and to progressive neurological dysfunction [2–5].

Here, we describe the history of two adolescent sisters who presented with cardiac arrest after consuming minimal amounts of alcohol. We underline the diagnostic pitfalls in the rapidly expanding multi-gene and whole-exome sequencing approaches until a definite diagnosis was established, which meant a big change in perspective for the family and the cardiologists in charge.

## Case report

**Fig. 1:**
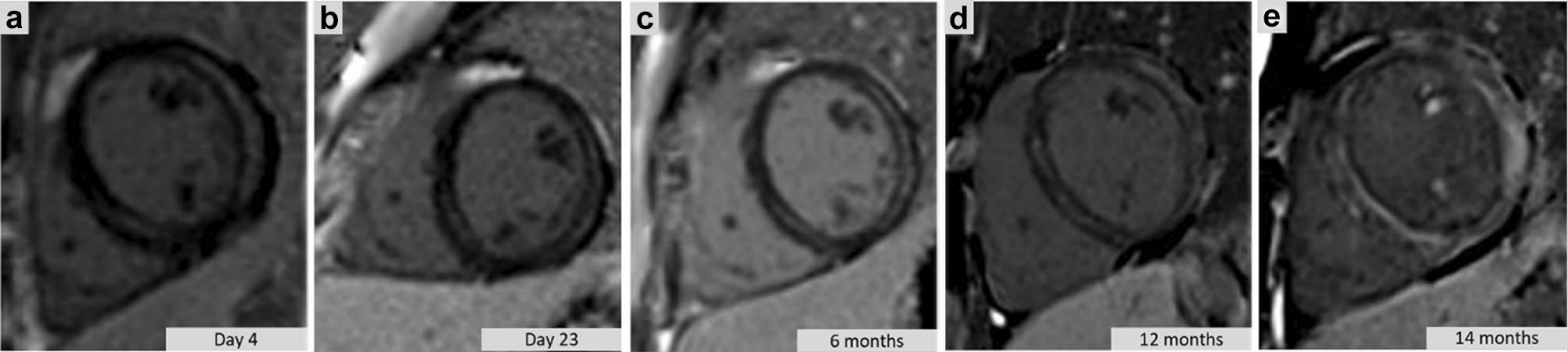
**a–e** Serial cardiac magnetic resonance images of patient 1 at different times after the first cardiac arrest revealing subepicardial late gadolinium enhancement (details in text)

The family originated from the same region in Tyrol, and parental consanguinity was denied. The first catastrophic event occurred in the older previously healthy girl (patient 1) at age 14 years. She had previously experienced an upper respiratory tract infection, treated symptomatically. She was found with vomit and cardiac arrest in bed after returning from a New Year’s Eve celebration, where she consumed only one Radler Beer (low alcohol mixed beer). She survived cardiopulmonary resuscitation and two shocks of the automated external defibrillator, which detected ventricular tachycardia and ventricular fibrillation before reaching sinus rhythm. Further examinations took place at the emergency department of the Medical University of Innsbruck. Electrocardiogram (ECG) showed an enlarged P in lead II and an incomplete right branch block (sinus rhythm 81/min, P: 138 ms, PQ: 160 ms, QRS: 92 ms, QT: 330 ms QTc: 430 ms). Echocardiography displayed a reduced left ventricular function (fractional shortening 23%) and mitral insufficiency grade II. She presented elevated cardiac biomarkers, leucocytosis with neutrophilia, and positive serology for *Chlamydia* spp. and *Chlamydophila pneumoniae*. Screening for drug intoxications in plasma was negative, including ethyl alcohol (NAD/ADH) < 0.1 g/l. Cardiac magnetic resonance imaging (CMR) revealed subepicardial late gadolinium enhancement (LGE) of the lateral wall and midwall LGE of the inferior septum to a lesser extent, both associated with regional myocardial edema and overall preserved left ventricular systolic function, findings suggestive of myocarditis (Fig. 1a). Subsequent ECG showed a discrete ST-segment elevation in leads V2 and V3. Further cardiac examination within the next 6 months showed normal long-term ECG and echocardiographic normalization of left ventricular function (ejection fraction (EF) 40–56%). The ajmaline test and coronary angiography were unremarkable. Endomyocardial biopsies reported chronic myocarditis (borderline myocarditis according to the Dallas terminology) without myocardial fibrosis. Two closely repeated CMR scans 3 weeks and 6 months after the index event (Fig. 1b, c) showed shrinkage of the initial LGE areas with resolution of the myocardial edema. However, further CMR scans 12 and 14 months (Fig. 1d, e) after the 1st cardiac arrest revealed a clear disease progression with extensive circumferential LGE in a subepicardial and midwall pattern, accompanied with a globally diffuse myocardial edema and deterioration in systolic left ventricular function to an EF of 46%. Serology for *Chlamydia pneumoniae* remained equal, leading to the suspicion of a chronic active infection, for which the patient was treated with doxycycline. An NGS-Panel for Arrhythmias (48 genes; performed at the Department of Clinical Genetics, University of Amsterdam in 2015) detected two heterozygous variants of uncertain significance (class 3 variants): c.647C > T; (p.Ser216Leu) *SCN5A* and c.3374C > T; (p.Ala1125Val) in *CACNA1D*.

Unexpectedly, patient 1 survived another cardiac arrest a year later, once more in the context of an upper respiratory tract infection and after drinking low levels of alcohol. Cardiac examination included an angiography and repetition of an endomyocardial biopsy. A subcutaneous implantable cardioverter-defibrillator (S-ICD Boston Scientific) was provided under the diagnosis of progressive chronic myocarditis. Regular cardiac follow-up showed slow progression of the disease, with a dilated left ventricle (M-mode: left ventricular end-diastolic dimension 59.8 mm, and indexed left ventricular end-diastolic dimension 32.15) with reduced left ventricular function (fractional shortening 19.7%), in addition to mitral insufficiency grades II–III (type III b). Neither ECG nor the subcutaneous implantable cardioverter-defibrillator showed any arrhythmias or shocks, QT interval remained also normal. Clinically, the patient had no physical limitations.

Three years later, the younger sister (patient 2) died suddenly at the age of 15 during sleep after spending time with friends in the evening (consumption of 1–2 beers) and “feeling sick”. Autopsy of the heart revealed acute myocarditis without structural alterations. Toxicological exams showed blood alcohol levels of 0.13 permille. Genetic segregation analysis confirmed the *SCN5A* and *CACNA1D* variants in blood samples of the deceased daughter and her healthy mother. Under the possibility of an autosomal dominantly inherited maternal risk factor, there was a remarkable family burden as to whether who would be the next in line for cardiac arrest.

Over time, patient 1 underwent further cardiac controls. Since there was consistent evidence of a left-atrial and ventricular dilatation with EF below normal (48%), we considered further genetic investigation as necessary and performed a duo-exome including both sisters. This time, a homozygous mutation in the *PPA2*-Gene c.683C > T (p.Pro228Leu) was found and confirmed autosomal recessive PPA2-related mitochondriopathy. There was no neurological dysfunction in patient 1 when last seen at age 21 years.

## Discussion

PPA2-related disease is a rare condition comprising about 30 published families worldwide. Functional studies of PPA2 attributed a role for mitochondrial function and maintenance of the mitochondrial genome [3, 5, 6]. Experiments on yeast cultures revealed that the disruption of PPA2 still shows viable cell growth, which ceases immediately on respiratory carbon sources like ethanol [6]. Mutations leading to a severe reduction of PPA2 activity results in early onset mitochondriopathy with lactic acidosis, seizures, muscular hypotonia and cardiac arrhythmia leading to an early death, commonly within the first 2 years of life [4, 5]. Our patients and others with a higher residual PPA2 activity present in youth or young adulthood with an isolated cardiac expression, myocardial fibrosis, ventricular dilatation, and arrhythmias. Astoundingly, minimal amounts of alcohol act as a trigger that disrupts the function of the vulnerable heart of these patients, leading to cardiac arrest. Noteworthy, inflammation of the myocardium was frequently diagnosed, similar to our patients. It was stated that myocardial fibrosis was a consistent finding and might help to discriminate PPA2-related cardiomyopathy from viral myocarditis [4]. Progressive neurological signs were observed in few patients who survived their cardiac events, consisting of distal muscle weakness along with peripheral axonal neuropathy, external ophthalmoplegia, ptosis, ataxia, and spasticity [4].

Kennedy et al. reported a family with compound heterozygous mutations p.(Glu172Lys) and p.(Pro228 Leu) of *PPA2*, whose individuals showed severe cardiac symptoms after consuming less than 0.1 g of alcohol or sudden cardiac death after only one standard beer [2]. Viral infections may act as similar triggers [2]. The mutation c.683C > T, p.(Pro228Leu) results in a partial (25%) reduction of PPA2 activity [2], and it was found for the first time in a homozygous state in our patients. Despite denying consanguinity, the parents of our patients came from the same Tyrolian valley with a population of 3700 people, which points towards a founder mutation and highlights the importance of a detailed family history in case of an assumed genetic condition.

In our family, the first diagnostic tier was an NGS-Panel for Arrhythmias (48 genes), which detected two heterozygous variants of uncertain significance in *SCN5A* and *CACNA1D*. At this point, the PPA2*-*related disease had not been reported. Only through exome sequencing the diagnosis of the recently discovered mitochondriopathy was established. To conclude, our case report demonstrates how important it is to question inconclusive genetic results. It cannot be underestimated how significant it was for the family to know the correct diagnosis and to be relieved from the genetic burden of an autosomal dominantly inherited predisposition for cardiac arrest.

Moreover, we aim to increase awareness for rare diseases like the PPA2-related mitochondriopathy. An accurate diagnosis is important for medical care (cardiac and neurological surveillance) and for everyday life, specifically because alcohol intake can result in cardiac arrest and should be strictly avoided.

## Data Availability

Study data are available upon request

## Abbreviations

CMR: Cardiac magnetic resonance imaging
EF: Ejection fraction
ECG: Electrocardiogram
LGE: Late gadolinium enhancement
NGS: Next-generation sequencing
PPA2: Pyrophosphatase type 2
